# Exertional Rhabdomyolysis Among Active Component Members of the U.S. Armed Forces, 2019–2023

**Published:** 2024-04-20

**Authors:** Armed Forces Health Surveillance Division Defense Health Agency, US

## Abstract

A largely preventable condition, exertional rhabdomyolysis persists as an occupational hazard of military training and operations, especially in high heat environments among individuals exerting themselves to their physical endurance limits. During the 5-year surveillance period of this study, unadjusted incidence rates of exertional rhabdomyolysis per 100,000 person-years among U.S. active component service members fluctuated, reaching a low of 38.0 cases in 2020 and peaking at 40.5 cases in 2023. The rate in 2020 constituted a decline of 3.8% from the rate in 2019 (39.5 cases). Beginning in 2020, incidence rates per 100,000 person-years gradually increased, by 1.8% in 2021 (38.7 cases), 5.3% in 2022 (40.0 cases), and 6.6% in 2023 (40.5 cases). Consistent with prior reports, subgroup-specific crude rates in 2023 were highest among men, those less than 20 years old, non-Hispanic Black service members, Marine Corps or Army members, and those in combat-specific and ‘other’ occupations. Recruits experienced the highest rates of exertional rhabdomyolysis during each year, with incidence rates 6 to 10 times greater than all other service members.

**What are the new findings?:**

The 529 reported incident cases of exertional rhabdomyolysis among active component U.S. service members in 2023 represent an unadjusted annual incidence rate of 40.5 cases per 100,000 person-years, the highest rate observed during this study's 2019–2023 surveillance period. This increase in crude incidence rates was most noticeable in the Marine Corps, which reported a 10.5% rise in 2023, from the previous year. The rates in the Army remained steady for the last 2 years of the surveillance period, but have increased by 26.9% from 2019. This year’s report includes exertional rhabdomyolysis rates for the Coast Guard.

**What is the impact on readiness and force health protection?:**

Service members who experience exertional rhabdomyolysis may be at risk for recurrence, which could limit their military effectiveness and potentially predispose them to serious injury. The risk of exertional rhabdomyolysis can be reduced through prompt recognition of its symptoms by commanders, informed by awareness of environmental conditions, cognizance of troop fitness levels, emphasis on graded, individual preconditioning prior to more strenuous training, and adherence to recommended work and rest ratios featuring appropriate hydration schedules, especially in hot, humid weather.

## 1 BACKGROUND

Initiation of high-intensity physical activity at unaccustomed intensity or duration, particularly under heat stress, increases the risk of exertional rhabdomyolysis.^1^ A potentially serious condition, exertional rhabdomyolysis requires vigilance for early diagnosis and aggressive treatment to prevent severe consequences.

Rhabdomyolysis is characterized by the breakdown of skeletal muscle cells and leakage of intracellular contents (myoglobin, sarcoplasmic proteins, and electrolytes) into the extracellular fluid and the circulatory system. Myoglobin is toxic to the tubular cells of the kidney and can lead to renal failure. Rhabdomyolysis severity ranges from asymptomatic or mild elevation of serum muscle enzyme levels to life-threatening conditions due to electrolyte imbalances, acute kidney failure, disseminated intravascular coagulation, compartment syndrome, cardiac arrhythmia, and liver dysfunction.^1,2,3,4^

The characteristic triad of rhabdomyolysis symptoms are muscle pain, weakness and red to brown colored urine due to high levels of myoglobin, although over half of patients do not have all of these specific symptoms.^5^ The standard diagnostic criteria for exertional rhabdomyolysis are elevated serum creatine phosphokinase (CPK) levels indicating myonecrosis (usually defined as CPK level of at least 5 times the upper limit of normal) following recent exercise.^2,3,6^

Exertional rhabdomyolysis is most commonly identified among new recruits at recruit training and combat installations during the first 90 days of basic training,^7,8^ but can also be observed in athletes accustomed to intense training,^9^ particularly when they extend themselves to endurance limits.^10^ A history of heat illness and prior heat stroke have also been described as significant risk factors for recruits who sustained rhabdomyolysis,^8,11^ revealing the potential for comorbid conditions.

*MSMR* annually summarizes the numbers, rates, trends, risk factors, and locations of exertional heat injury occurrences including exertional rhabdomyolysis. This report assesses Military Heath System (MHS) data from 2019 through 2023.

Additional information about the definition, causes, and prevention of exertional rhabdomyolysis can be found in previous issues of *MSMR*.^7^

## 2 METHODS

The surveillance period ranged from January 2019 through December 2023 and included all individuals who served in the active components of the Army, Navy, Air Force, Marine Corps, Coast Guard, and Space Force (which was assigned to the Air Force for analysis purposes). All data used to determine incident exertional rhabdomyolysis diagnoses were derived from records routinely maintained in the Defense Medical Surveillance System (DMSS). These records document both ambulatory encounters and hospitalizations of active component members of the U.S. Armed Forces in fixed military and civilian (if reimbursed through the MHS) hospitals and clinics worldwide. In-theater diagnoses of exertional rhabdomyolysis were identified from medical records of service members deployed to Southwest Asia and the Middle East whose health care encounters were documented in the Theater Medical Data Store.

A case of exertional rhabdomyolysis was defined as an individual with International Classification of Diseases, 9th and 10th revisions (ICD-9/ICD-10) diagnostic codes in any position indicating a hospitalization or outpatient medical encounter with either “rhabdomyolysis” or “myoglobinuria,” with a diagnosis in any position of 1 of the following: “volume depletion (dehydration),” “effects of heat and light,” “effects of thirst (deprivation of water),” “exhaustion due to exposure,” or “exhaustion due to excessive exertion (overexertion)”^12^ (**Table 1**). Each individual could be considered an incident case of exertional rhabdomyolysis only once per calendar year.

Cases of rhabdomyolysis associated with trauma, intoxication, and adverse drug reactions were excluded.^6^ For health surveillance purposes, recruits were identified as active component members assigned to service-specific training locations during coincident service-specific basic training periods. Recruits were considered a separate category of enlisted service members in summaries of exertional rhabdomyolysis by military grade overall.

In-theater diagnoses of exertional rhabdomyolysis were analyzed separately using the same case-defining criteria and incidence rules that identified incident cases at fixed treatment facilities. Records of medical evacuations from the U.S. Central Command (CENTCOM) area of responsibility (AOR) (i.e., Southwest Asia, Middle East) to a medical treatment facility outside the CENTCOM AOR were analyzed separately. Evacuations were considered case-defining if affected service members met the aforementioned criteria in a permanent military medical facility in the U.S. or Europe from 5 days preceding until 10 days following their evacuation dates.

## 3 RESULTS

In 2023, there were 529 cases of rhabdomyolysis likely associated with physical exertion or heat stress (i.e., exertional rhabdomyolysis), with 41.6% (n=220) resulting in hospitalization (**Table 2**). Consistent with prior annual reports, crude incidence rates remained highest among men, those younger than 20 years old, non-Hispanic Black service members, Marine Corps or Army members, and those in ‘other’ and combat-specific occupations. Recruits continued to present the highest rates of exertional rhabdomyolysis in 2023, at a rate of over 12 times greater than officers and other enlisted service members.

**Figure 1:**
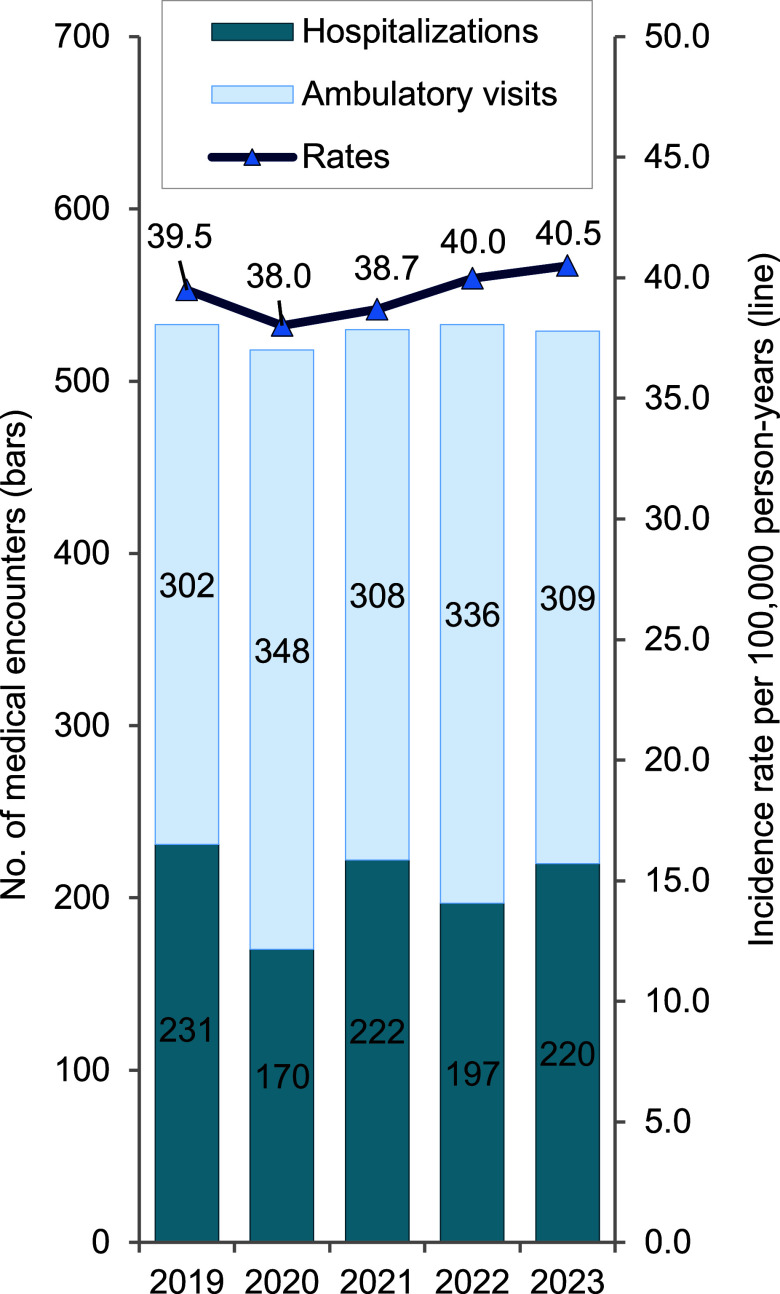
Incident Cases and Incidence Rates of Extertional Rhabdomyolysis, by Source of Report and Year of Diagnosis, Active Component, U.S. Armed Forces, 2019–2023

During the 5-year surveillance period, total crude incidence rates of exertional rhabdomyolysis per 100,000 person-years (p-yrs) among U.S. active component service members (ACSMs) fluctuated from a low of 38.0 cases in 2020 and high of 40.5 cases in 2023. The rate in 2020 constituted a decline of 3.8% from the 2019 rate (39.5 cases). Beginning in 2020, however, the total crude incidence rates per 100,000 p-yrs began gradually increasing: 1.8% in 2021 (38.7 cases), 5.3% in 2022 (40.0 cases), and 6.6% in 2023 (40.5 cases) (**Figure 1**).

**Figure 2:**
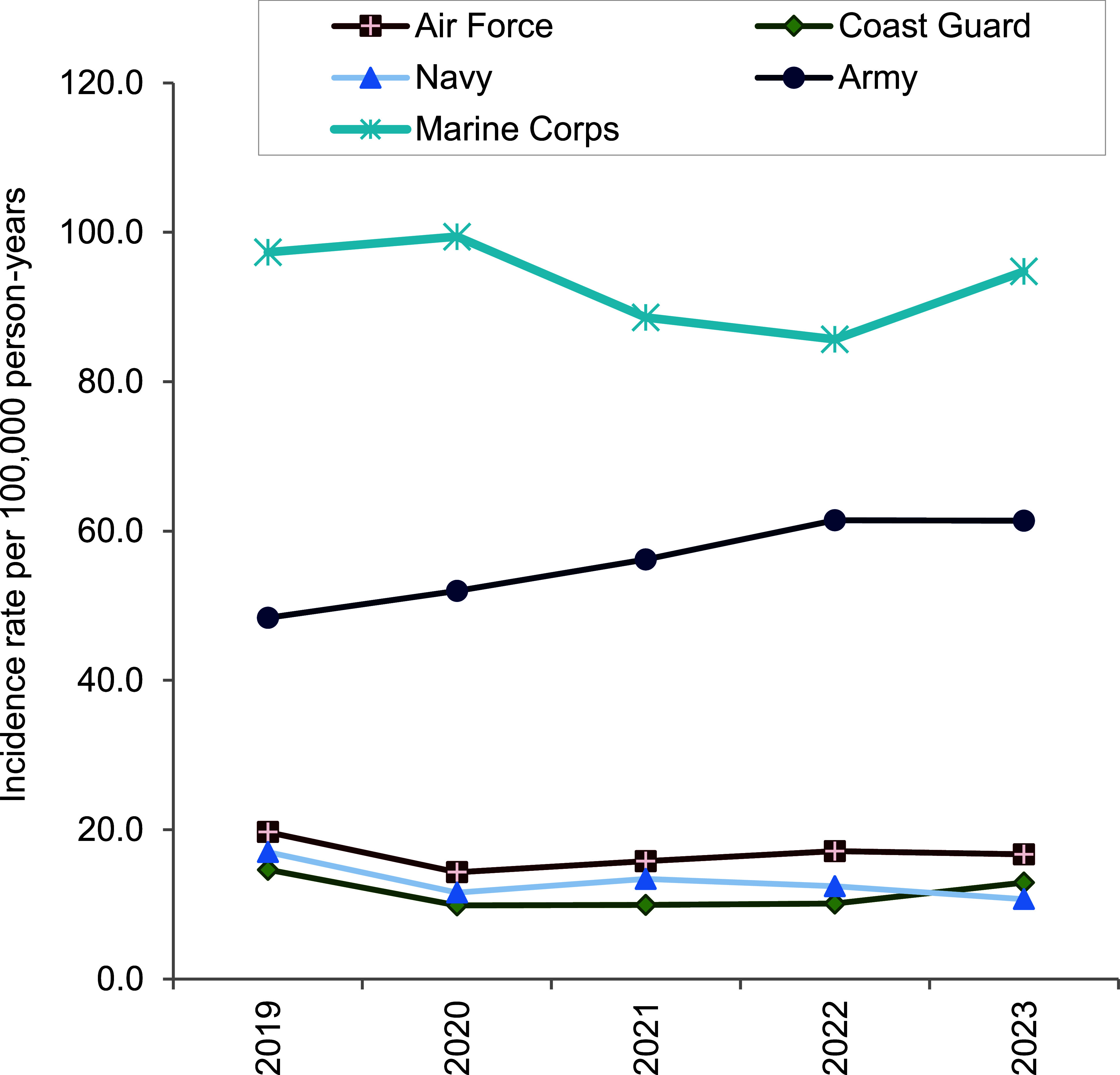
Annual Incidence Rates of Exertional Rhabdomyolysis, by Service, Active Component, U.S. Armed Forces, 2019–2023

The military service branch with the highest rate was the Marine Corps, at 94.7 cases per 100,000 p-yrs, followed by the Army, at 61.4; Air Force, at 16.7; Coast Guard, at 12.9; with the lowest rate observed in the Navy, at 10.7 cases per 100,000 p-yrs (**Figure 2**). Significant variability was observed within services. The Marine Corps displayed wide fluctuations, with a notable increase of 10.5% in 2023 compared to 2022. Trends in the Army showed more consistency, steadily increasing from 48.4 cases in 2019 to 61.4 cases per 100,000 p-yrs in both 2022 and 2023, representing a 26.9% increase over the 5-year period. Rates in the Air Force, Navy, and Coast Guard generally remained all below 20 cases per 100,000 p-yrs, albeit with occasional greater annual fluctuations in range.

**Figure 3:**
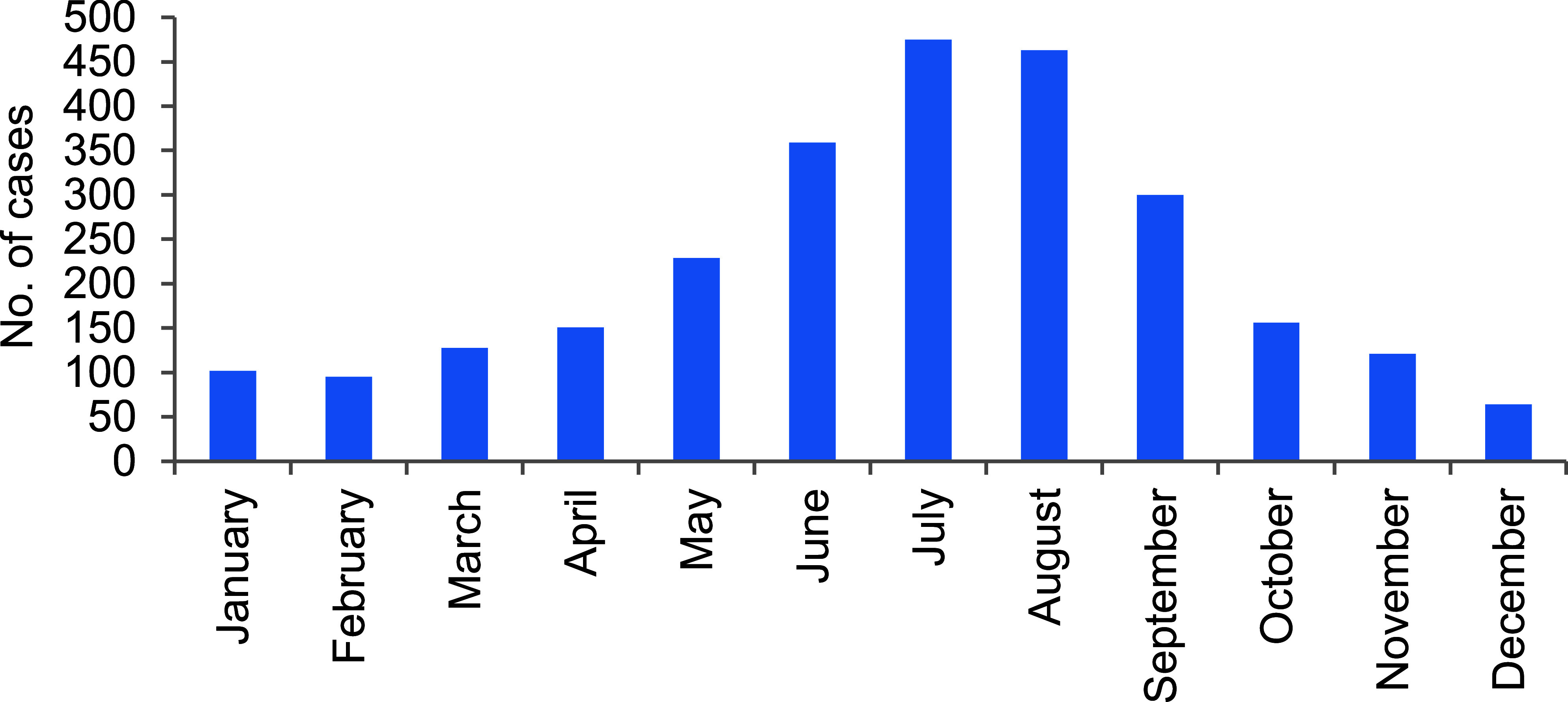
Cumulative Numbers of Exertional Rhabdomyolysis Cases, by Month of Diagnosis, Active Component, U.S. Armed Forces, 2019–2023

Hospitalization rates were lowest in 2020, at 32.8%, with an average of 39.4% over the 5-year period (**Figure 1**). From 2019 to 2023, approximately three-quarters (74.8%) of cases occurred during the warmer months (i.e., April through September) (**Figure 3**).

**Rhabdomyolysis by location**

During the 5-year surveillance period, 12 installations reported at least 50 cases each; when combined, these installations diagnosed more than half (56.3%) of all cases (**Table 3**). Four of those 12 installations support recruit or basic combat training centers: Marine Corps Recruit Depot (MCRD) Parris Island, SC; Fort Moore, GA; Joint Base San Antonio-Lackland, TX; and Fort Leonard Wood, MO; while 6 of those installations support large combat troop populations: Fort Liberty, NC; MCB Camp Lejeune/Cherry Point, NC; Marine Corps Base (MCB) Camp Pendleton, CA; Fort Cavazos, TX; Fort Shafter, HI; and Fort Campbell, KY. From 2019 to 2023, MCRD Parris Island and Fort Liberty together accounted for about one-fifth (20.3%) of all cases (**Table 3**).

**Rhabdomyolysis in Iraq and Afghanistan**

Eight cases of exertional rhabdomyolysis were diagnosed and treated in Iraq and Afghanistan during the surveillance period; 2 were diagnosed in 2019, and 1 case annually from 2020 to 2022, and 3 cases in 2023 (data not shown). The majority of those deployed service members affected by exertional rhabdomyolysis were male (n=5), non-Hispanic White (n=4) or non-Hispanic White (n=3), in the Army (n=5), and enlisted (n=6). One ACSM was medically evacuated for exertional rhabdomyolysis and dehydration in January 2023 (data not shown).

## 4 DISCUSSION

This report presents findings that indicate total crude incidence rates of exertional rhabdomyolysis remained relatively stable between 2019 and 2023, ranging from 38.0 to 40.5 cases per 100,000 p-yrs. The lowest rate was observed in 2020, coinciding with the height of COVID-19 pandemic restrictions. Rates began to rise thereafter, reaching a peak increase of 6.6% in 2023 in comparison to the nadir year of 2020.

Exertional rhabdomyolysis continues to occur most frequently from mid-spring through early fall in the Northern Hemisphere, at installations that support basic combat and recruit training or major Army and Marine Corps combat units. Recruits can be exposed to environmental situations that require acclimatization to high heat and humidity during the warmer months, while Soldiers and Marines in combat units often perform rigorous unit physical training, personal fitness training, and field training exercises regardless of weather conditions.

The annual incidence rates for exertional rhabdomyolysis among non-Hispanic Black service members were higher, by approximately 1.8 times, than the rates among members of other races and ethnicities. This observation has been attributed, at least in part, to an increased risk of exertional rhabdomyolysis among individuals with sickle cell trait (SCT),^13,14,15,16^ for which the carrier frequency is approximated at 1 in 13 non-Hispanic Blacks in the U.S.^17^ A significant association between SCT and the risks of exertional rhabdomyolysis is supported by studies among U.S. service members.^18,19^ The rhabdomyolysis-related deaths of 2 SCT-positive service members (an Air Force member and Navy recruit) in 2019 after physical training stress this potential risk.^20,21^ Although previous studies showed that SCT was associated with a 54% increase in exertional rhabdomyolysis risk, no similar association was found with risk of death. According to some experts, however, these studies missed deaths due to exertional sickling, and controversies with defining exertional rhabdomyolysis, its associations with disease progression and severity, and its prevention and management evince the need for further research.^22,23^ Nevertheless, changes to the 2023 TRADOC Regulation include adding “sickle cell trait as a risk factor” and updated recommendations for screening, early recognition and prevention of exercise collapse associated with sickle cell trait (ECAST).^24^

The findings of this report should be interpreted with consideration of its limitations. A diagnosis of rhabdomyolysis alone does not indicate cause. Ascertaining the probable causes of exertional rhabdomyolysis cases was attempted by utilizing a combination of ICD-9/ICD-10 diagnostic codes related to rhabdomyolysis with additional codes indicating effects of exertion, heat, or dehydration. Other ICD-9/ICD-10 codes were used to exclude cases of rhabdomyolysis that may have been secondary from trauma, intoxication, or adverse drug reactions.

Recruits were identified using an algorithm based on age, rank, location, and time in service, which was only an approximation and likely resulted in some misclassification of recruit training status.

Management after treatment for exertional rhabdomyolysis, including the decision to return to physical activity and duty, is a persistent challenge for both athletes and military members.^21^ Service members who experience a clinically-confirmed exertional rhabdomyolysis event should be further evaluated and risk-stratified for recurrence before return to activity or duty.^6,25,26^ Service-specific guidelines may require temporary or permanent duty restriction following rhabdomyolysis, as recently diagnosed individuals remain at a higher risk for future heat illnesses.

The most severe consequences of exertional rhabdomyolysis are preventable with effective mitigation measures and heightened suspicion of probability when environmental conditions favor muscular injury. Commanders and supervisors at all levels should ensure that guidelines for heat illness prevention are consistently implemented, maintain vigilance for early signs of exertional heat injury, and intervene aggressively when exertional rhabdomyolysis is suspected.^1^

## Figures and Tables

**Table 1:** ICD-9 and ICD-10 Diagnostic Codes Utilized to Define Cases of Exertional Rhabdomyolysis

**Primary condition**	**ICD-9^a^**	**ICD-10^a^**
Rhabdomyolysis	728.88	M62.82
Myoglobinuria	791.3	R82.1
**Associated conditions**	**ICD-9^a^**	**ICD-10^a^**
Volume depletion (dehydration)	276.5^*^	E86.0, E86.1, E86.9
Effects of heat and light	992.0-992.9	T67.0^*^-T67.9^*^
Effects of thirst (deprivation of water)	994.3	T73.1^*^
Exhaustion due to exposure	994.4	T73.2^*^
Exhaustion due to excessive exertion (overexertion)	994.5	T73.3^*^

Abbreviation: ICD, International Classification of Diseases, 9th and 10th revisions.

^a^An asterisk (*) indicates that any subsequent digit or character is included.

**Table 2:** Incident Cases^a^ and Incidence Rates^b^ of Exertional Rhabdomyolysis, Active Component, U.S. Armed Forces, 2023

	**Hospitalizations**		**Ambulatory Visits**		**Total**	
	No.	Rate^b^	No.	Rate^b^	No.	Rate^b^
**Total**	220	16.8	309	23.7	529	40.5
** Gender**						
Male	209	19.4	286	26.6	495	46.0
Female	11	4.8	23	10.0	34	14.8
** Age group**, y						
<20	34	22.8	87	58.3	121	81.1
20-24	77	23.3	92	27.9	169	51.2
25-29	60	19.6	78	25.4	138	45.0
30-34	33	15.2	37	17.0	70	32.2
35-39	7	4.2	10	6.0	17	10.1
40+	9	6.7	5	3.7	14	10.4
** Race and ethnicity**						
White, non-Hispanic	105	15.2	149	21.6	254	36.8
Black, non-Hispanic	55	26.5	66	31.8	121	58.4
Hispanic	34	13.6	67	26.8	101	40.4
Other/unknown	26	16.3	27	16.9	53	33.2
** Service**						
Army	129	28.8	146	32.6	275	61.4
Navy	16	4.9	19	5.8	35	10.7
Air Force	28	8.7	26	8.0	54	16.7
Marine Corps	45	26.6	115	68.1	160	94.7
Coast Guard	2	5.2	3	7.7	5	12.9
** Military status**						
Enlisted	167	16.1	191	18.4	358	34.4
Officer	24	9.8	49	20.1	73	29.9
Recruit	29	124.6	69	296.4	98	421.0
** Military occupation**						
Combat-specific^c^	70	42.0	85	51.0	155	92.9
Motor transport	8	18.4	5	11.5	13	29.9
Pilot/air crew	2	4.4	0	0.0	2	4.4
Repair/engineering	22	5.9	32	8.6	54	14.6
Communications/intelligence	36	12.9	42	15.1	78	28.0
Health care	19	18.0	9	8.5	28	26.5
Other/unknown	63	21.3	136	46.0	199	67.4
** Home of record**						
Midwest	26	12.7	49	24.0	75	36.8
Northeast	26	16.5	43	27.4	69	43.9
South	111	19.7	147	26.1	258	45.8
West	48	15.9	58	19.2	106	35.1
Other/unknown	9	11.3	12	15.1	21	26.4

Abbreviations: No., number; y, years.

^a^One case per person per year.

^b^Rate per 100,000 person-years.

^c^Infantry/artillery/combat engineering/armor.

**Table 3:** Incident Cases of Exertional Rhabdomyolysis by Installation (with at least 20 cases during the period), Active Component, U.S. Armed Forces, 2019–2023

**Location of Diagnosis**	**No.**	**% Total**
Fort Liberty, NC	288	10.9
MCRD Parris Island, SC	248	9.4
Fort Moore, GA	199	7.5
MCB Camp Lejeune/Cherry Point, NC	134	5.1
MCB Camp Pendleton, CA	99	3.7
Fort Campbell, KY	92	3.5
Fort Cavazos, TX	84	3.2
JBSA-Lackland AFB, TX	78	3.0
NMC San Diego, CA	77	2.9
Fort Johnson, LA	500	4.0
Fort Shafter, HI	75	2.8
Fort Leonard Wood, MO	64	2.4
MCB Quantico, VA	50	1.9
Fort Johnson, LA	48	1.8
Fort Carson, CO	48	1.8
Fort Bliss, TX	44	1.7
NH Okinawa, Japan	38	1.4
NH Twentynine Palms, CA	33	1.2
Fort Belvoir, VA	31	1.2
NMC Portsmouth, VA	26	1.0
Fort Eisenhower, GA	22	0.8
Fort Riley, KS	21	0.8
Other/unknown locations	844	31.9
Total	2,643	100.0

Abbreviations: No., number; MCRD, Marine Corps Recruit Depot; MCB, Marine Corps Base; NMC, Naval Medical Center; JBSA, Joint Base San Antonio; NH, Naval Hospital.

## AUTHOR AFFILIATIONS

Defense Health Agency, U.S. Department of Defense
